# Design and synthesis of PDSPTCF as an influential Brønsted-Lewis acidic catalyst for the producing benzo[*a*]benzo[6,7]chromeno[2,3-*c*]phenazines

**DOI:** 10.1038/s41598-024-78824-2

**Published:** 2024-12-02

**Authors:** Wade Ghribi, Muhsen Al-ibadi, Subbulakshmi Ganesan, M. Ravi Kumar, Yashwantsinh Jadeja, Jasgurpreet Singh Chohan, Mamata Chahar, Rajni Verma, I. B. Sapaev, Abhinav Kumar

**Affiliations:** 1 Department of Computer Engineering, College of Computer Science, King Khalid University, Al-Faraa, Kingdom of Saudi Arabia; 2 Department of Chemistry, College of Science, University of Kufa, Najaf, Iraq; 3 Medical Laboratory Technique College, the Islamic University, Najaf, Iraq; 4 Department of Chemistry and Biochemistry, School of Sciences, JAIN (Deemed to Be University), Bangalore, Karnataka India; 5 Department of Chemistry, Raghu Engineering College, Visakhapatnam, Andhra Pradesh 531162 India; 6 Department of Chemistry, Faculty of Science, Marwadi University Research Center, Marwadi University, Rajkot, Gujarat 360003 India; 7 School of Mechanical Engineering, Rayat Bahra University, Kharar, Punjab 140103 India; 8 Faculty of Engineering, Sohar University, PO Box 44, Sohar, PCI 311 Oman; 9 Department of Chemistry, NIMS Institute of Engineering and Technology, NIMS University Rajasthan, Jaipur, India; 10 Department of Applied Sciences, Chandigarh Engineering College, Chandigarh Group of Colleges-Jhanjeri, Mohali, Punjab Pin 140307 India; 11 Head of the department «Physics and Chemistry», “Tashkent Institute of Irrigation and Agricultural Mechanization Engineers” National Research University, Tashkent, Uzbekistan; 12 Scientific Researcher, University of Tashkent for Applied Sciences, Str. Gavhar 1, 100149 Tashkent, Uzbekistan; 13 Scientific Researcher, Western Caspian University, Baku, Azerbaijan; 14 Department of Nuclear and Renewable Energy, Ural Federal University Named After the First President of Russia Boris Yeltsin, Ekaterinburg, Russia 620002; 15 Department of Mechanical Engineering, Karpagam Academy of Higher Education, Coimbatore, 641021 India

**Keywords:** Benzo[*a*]benzo[6,7]chromeno[2,3-*c*]phenazine, Multicomponent domino reactions (MDRs), 4,4'-(1,4-phenylene)di(sulfonic)pyridinium tetrachloroferrate (PDSPTCF), Brønsted-Lewis acidic catalyst, Organic–inorganic hybrid salt, Biocatalysis, Catalyst synthesis, Catalytic mechanisms, Heterogeneous catalysis, Organocatalysis

## Abstract

Initially, 4,4'-(1,4-phenylene)di(sulfonic)pyridinium tetrachloroferrate (PDSPTCF) as a novel organic–inorganic hybrid salt was synthesized and identified by elemental mapping, energy-dispersive X-ray spectroscopy, inductively coupled plasma atomic emission spectrometer, Raman spectroscopy, Fourier-transform infrared spectroscopy, X-ray diffraction, field emission scanning electron microscopy, vibrating-sample magnetometry, and thermal gravimetric (TG) techniques. Then, the catalytic performance of this hybrid salt was assessed for the producing benzo[*a*]benzo[6,7]chromeno[2,3-*c*]phenazine derivatives via one-pot multicomponent domino reaction (MDR) of benzene-1,2-diamine, 2-hydroxynaphthalene-1,4-dione and aldehydes under optimal conditions (70 °C, solvent-free, 5 mol% PDSPTCF) in short reaction times and high yields. Highly efficacy of the PDSPTCF for the production of benzo[*a*]pyrano[2,3-*c*]phenazines can be assigned to the synergistic effect of Lewis and Brønsted acids, and having two positions of each acid (i.e., FeCl_4_ˉ and –SO_3_H). In addition, this catalyst showed good reproducibility with six cycles of recycling.

## Introduction

Structures consisting of organic and inorganic components with interaction on a molecular scale are generally defined as organic–inorganic hybrid materials^1,2^. The mentioned interaction can occur through weak forces such as van der Waals forces, hydrogen bonding, and electrostatic interactions or by strong ionic and covalent bonds^1–3^. It should be noted that properties of the hybrid compounds are not only sum of their inorganic and organic materials characteristics but also connected to the ratio of each part and the nature of the interfaces^4^. An important subgroup of these substances includes organic–inorganic hybrid salts^5^. Hybrid salts have several significant advantages, including simple separation from the process reactor, effectiveness, customizable for different applications, non-corrosiveness, environmentally friendly nature, and suitable thermal and chemical durability^2,3^. These materials have various usages in industrial and pharmaceutical fields; e.g., they have been employed in photo-detector^6^, sensors^7^, semiconductor^8^, optical devices^9^, luminescence studies^10^, cancer therapy^11^, fluid–fluid separations^12^, and in organic synthesis as effective catalysts^13–21^.

In modern synthesis, the progress of new synthetic procedures that offer maximum structural variation with step, pot, atom, and cost savings is occurring continuously^22^. Cascade (or domino) methods that generate several C-heteroatom and C–C bonds in one pot are particularly attracting attention, and they are of great importance in the effort to discover biologically active substances and drugs^23^. On the other hand, multicomponent reactions (MCRs) are useful in the field of producing heterocyclic scaffolds, especially the construction of diverse chemical libraries of "drug-like" molecules in one step, due to their valuable properties such as atom economy, favorable efficiency, short reaction time, and good stereo-control with simple purification operations^24–29^. Therefore, the integration of these two methods i.e., one-pot multicomponent domino reactions (MDRs), especially in the absence of solvent, have appeared as an efficacious technique^30^. Today, they have significantly impacted the field of synthetic transformations, especially in the production of heterocyclic compounds, and have demonstrated excellent overall yields, short reaction times, and good stereo-control with simple purification operation^31^.

Hence, the introduction of new MDRs for the synthesis of vital heterocycles with integrated structural roles in a wide range of bioactive molecules is an important issue for organic and medicinal chemists^31^. Heterocycles with functionalized oxygen and nitrogen play an essential role in medicinal chemistry^32–37^. Among them, heterocyclic systems with chromene and phenazine moieties are an important subgroup of aza-polycyclic scaffolds that exhibit various biological functions in synthetic and natural products, comprising antitumor, antimicrobial, anti-asthmatic, antimalarial, antifungal, anti-parasitic, antibiotic, anti-HIV (Human immunodeficiency virus), and antioxidant activities^38–47^. Also, due to their ability to intercalate their deoxyribonucleic acid (DNA), they show anti-cancer activity in tumors and solid leukaemia^46,48^. Pyridophenazinediones and pyridazinophenazinedione derivatives are well-known compounds in the mentioned field^46,48^. One group of these compounds is benzo[*a*]benzo[6,7]chromeno[2,3-*c*]phenazine derivatives, which despite the numerous applications of few catalysts for their production have been reported in the literature. L-proline^49^, Zr-guanine-MCM-41^50^, γ‐Fe_2_O_3_@SiO_2_‐SCH_2_CO_2_H^51^, Ni-Gly-isatin@boehmite^52^, and *p*-toluenesulfonic acid^53^ are among the available catalysts for this synthetic transformation. Protocols based on these catalysts, despite their advantages, suffer from drawbacks such as the pressing need for high temperatures or unconventional energy sources (e.g. microwave radiation)^49,50^, using large amounts of catalysts^53^, complex steps of catalyst synthesis^51^, prolonged reaction time^50–53^, low efficiency of some products^50^, tedious work-up procedures^49^, and the ineffectiveness of the catalyst in solvent-free environment acknowledging the important point that has been proven today, "the best solvent is solvent-free, and if a solvent (diluent) is required, it should preferably be H_2_O"^50,52,53^. Therefore, the presentation of new approaches that enable easy and fast synthesis of these derivatives is strongly felt in the literature.

Considering the above points and by combining the advantages of one-pot multicomponent domino reactions, solvent-free conditions reaction, benzo[*a*]benzo[6,7]chromeno[2,3-*c*]phenazines, and inorganic–organic hybrid materials, in this paper, a novel inorganic–organic hybrid salt, namely, 4,4'-(1,4-phenylene)di(sulfonic)pyridinium tetrachloroferrate (PDSPTCF) was synthesized and characterized. Next, it was used as an efficient Lewis– Brønsted acidic catalyst for the production of benzo[*a*]benzo[6,7]chromeno[2,3-*c*]phenazines via one-pot MDR of 2-hydroxynaphthalene-1,4-dione (2 eq.), benzene-1,2-diamine (1 eq.), and aldehydes (1 eq.) under solvent-free circumstances at 70 °C. The performance of our catalyst during this synthesis (**1a-12a**, 88% to 98%, 12 to 20 min) minimized many of the drawbacks of the previously mentioned protocols.

## Experimental

### Materials and apparatus

Information about all the materials and apparatus used are listed in the related files.

### PDSPTCF preparation

**Fig. 1:**
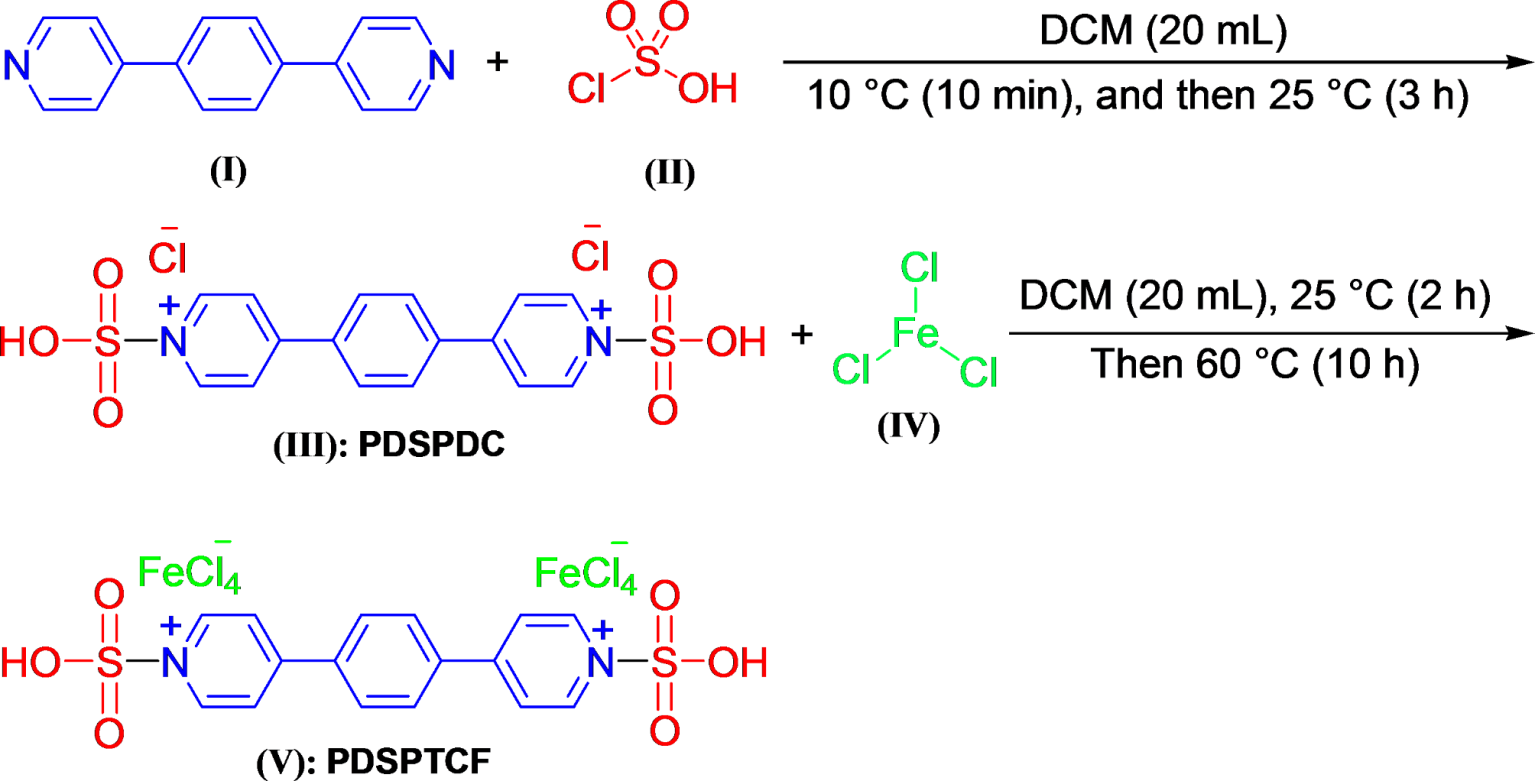
PDSPTCF preparation.

5 mmol of 1,4-bis(pyrid-4-yl)benzene **(I)** (0.232 g) in 20 mL dichloromethane (DCM) was added slowly to a solution of 10 mmol of chlorosulfonic acid **(II)** (1.165 g) in 20 mL DCM. The mixture was stirred at 10 °C for 10 min, and then at room temperature for 3 h. Subsequently, the DCM was evaporated; the residue was cleaned with 3 mL of petroleum ether (two times) and dried in vacuum at 90 °C to created 4,4'-(1,4-phenylene)di(sulfonic)pyridinium dichloride (**III:** PDSPDC). Lastly, 10 mmol of FeCl_3_ **(IV)** (1.622 g) was slowly added to 5 mmol of PDSPDC (2.325 g) and stirred for 2 h at 25 °C and 10 h at 60 °C to give 4,4'-(1,4-phenylene)di(sulfonic)pyridinium tetrachloroferrate (**V**: PDSPTCF) with 98% yield (Fig. 1). It should be noted that in both stages of the formation of compounds **IV** and **V**, the consumption of reactants were controlled through thin layer chromatography (*n*-hexane/ethyl acetate, 3:1, respectively).

### General process in the synthesis of benzo[a]benzo[6,7]chromeno[2,3-c]phenazines

**Fig. 2:**
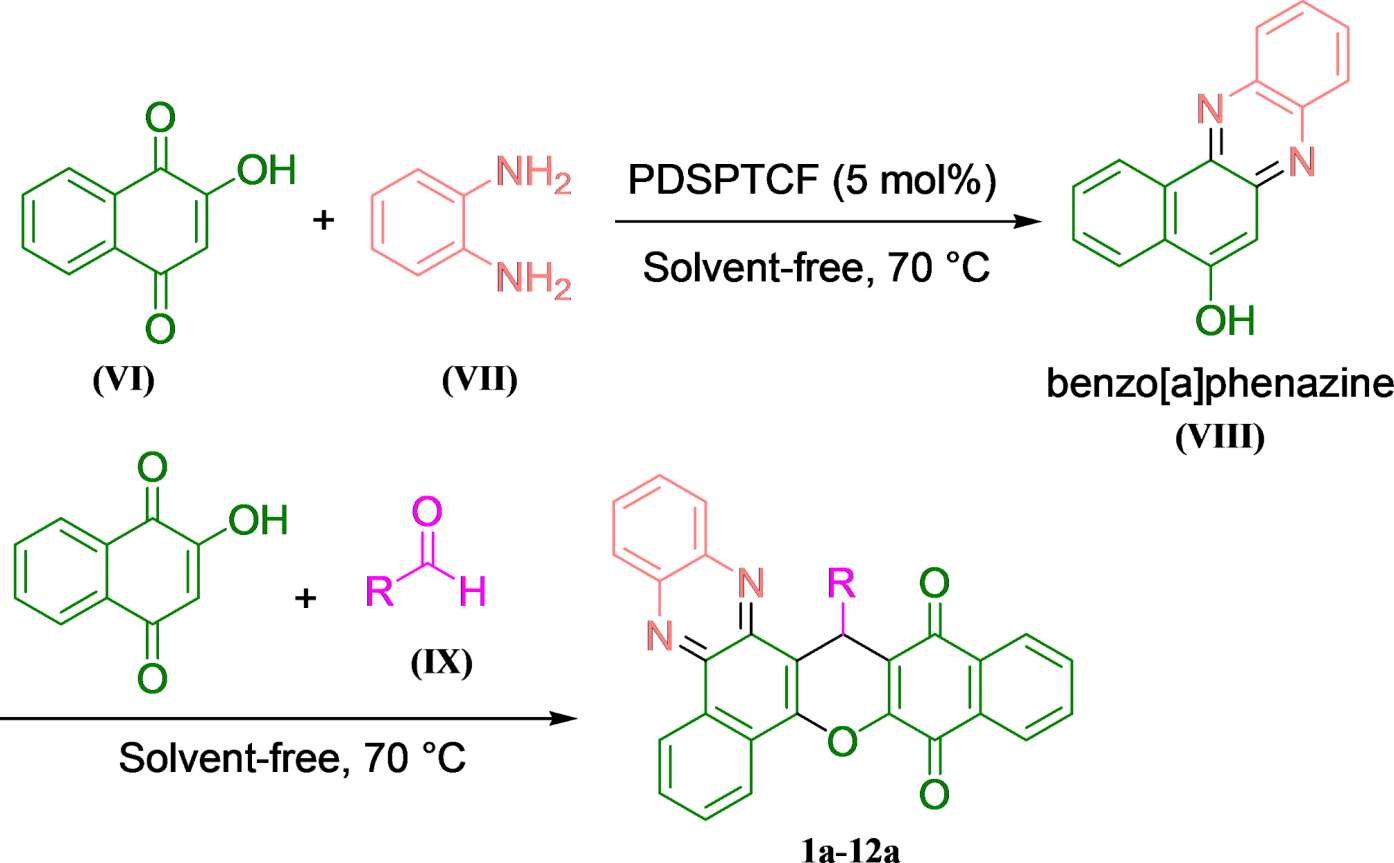
General process in the synthesis of benzo[*a*]benzo[6,7]chromeno[2,3-*c*]phenazine.

A mixture of 1 mmol 2-hydroxynaphthalene-1,4-dione **(VI)** (1.622 g), 1 mmol benzene-1,2-diamine **(VII)** (0.168 g) and 5 mol% PDSPTCF was stirred at 70° under a solvent-free domino reaction to afford benzo[*a*]phenazine **(VIII)**. After that, the second mole of **VI** and aldehyde **(IX)** (1 mmol) were added to **VIII** (this step: multicomponent reaction). The mixture was stirred until TLC confirmed the complete consumption of the reactants. The residue was then cooled to 25 °C and 12 mL of ethyl acetate (EtOAc) was added and the insoluble PDSPTCF was simply detached by centrifugation and decantation {the isolated hybrid salt was triturated with 3 mL EtOAc (two times) and dried for usage in the next run}. Ultimately, the pure benzo[*a*]benzo[6,7]chromeno[2,3-*c*]phenazines (**1a-12a**) were attained by recrystallization of the remainder solid in 95% ethanol (EtOH) after evaporation of EtOAc (Fig. 2).

### Spectroscopic data of some products

**Product 1a:** ^1^H NMR (500 MHz, DMSO-*d*_*6*_): δ (ppm) 5.60 (s, 1H, CH), 7.03 (t, *J* = 8.1, 1H, H_Ar_), 7.18 (d, *J* = 7.5, 1H, H_Ar_), 7.34–7.44 (m, 2H, H_Ar_), 7.76–8.26 (m, 10H, H_Ar_), 8.57 (d, *J* = 8.0, 1H, H_Ar_), 9.22 (d, J = 8.0, 1H, H_Ar_); ^13^C NMR (125 MHz, DMSO-*d*_*6*_): δ (ppm) 32.47, 113.43, 114.32, 116.15, 121.53, 122.80, 123.43, 128.09, 129.23, 130.00, 130.78, 131.23, 131.72, 132.39, 132.99, 133.47, 133.60, 134.10, 134.64, 135.27, 138.99, 139.73, 140.95, 143.38, 145.09, 146.92, 149.06, 157.50, 176.67, 178.03.

**Product 5a:** ^1^H NMR (500 MHz, DMSO-*d*_*6*_): δ (ppm) 6.00 (s, 1H, CH), 7.43 (t, J = 7.8, 1H, H_Ar_), 7.56 (t, J = 8.1, 1H, H_Ar_), 7.81–8.11 (m, 9H, H_Ar_), 8.29–8.35 (m, 1H, H_Ar_), 8.45 (s, 1H, HAr), 8.53–8.63 (m, 1H, H_Ar_), 8.82 (d, J = 8.1, 1H, H_Ar_), 9.28 (d, J = 8.1, 1H, H_Ar_) (Fig. S3); ^13^C NMR (125 MHz, DMSO-*d*_*6*_): δ (ppm) 31.01, 114.50, 115.40, 118.21, 119.10, 120.95, 121.93, 123.64, 125.38, 125.89, 126.43, 128.01, 128.48, 128.96, 129.65, 130.61, 135.11, 135.94, 138.31, 139.19, 140.03, 140.94, 143.35, 144.44, 146.89, 148.93, 154.11, 155.95, 176.11, 177.01.

**Product 11a:** ^1^H NMR (500 MHz, DMSO-*d*_*6*_): δ (ppm) 2.22 (s, 3H, CH_3_), 5.59 (s, 1H, CH), 7.00 (t, *J* = 7.7, 1H, H_Ar_), 7.11 (d, *J* = 8.1, 2H, H_Ar_), 7.33 (d, *J* = 8.1, 2H, H_Ar_), 7.44–7.52 (m, 1H, H_Ar_), 7.75 (t, *J* = 8.1, 1H, H_Ar_), 7.91–8.16 (m, 6H, H_Ar_), 8.37 (t, *J* = 8.1, 1H, H_Ar_), 8.45–9.28 (m, 1H, H_Ar_), 9.31 (t, *J* = 8.1, 1H, H_Ar_); ^13^C NMR (125 MHz, DMSO-*d*_*6*_): δ (ppm) 20.72, 34.17, 113.30, 114.67, 115.99, 116.62, 120.20, 121.47, 122.90, 124.04, 126.24, 128.12, 128.62, 129.19, 129.83, 130**.**70, 131.27, 131.96, 132.70, 133.28, 133.84, 134.72, 136.49, 138.31, 139.56, 140.43, 141.13, 145.53, 155.03, 155.90, 177.35, 178.22.

## Results and discussion

### Identification of PDSPTCF hybrid salt with physicochemical methods

In this section, the PDSPTCF skeleton was evaluated with the help of a set of physicochemical methods.

**Fig. 3:**
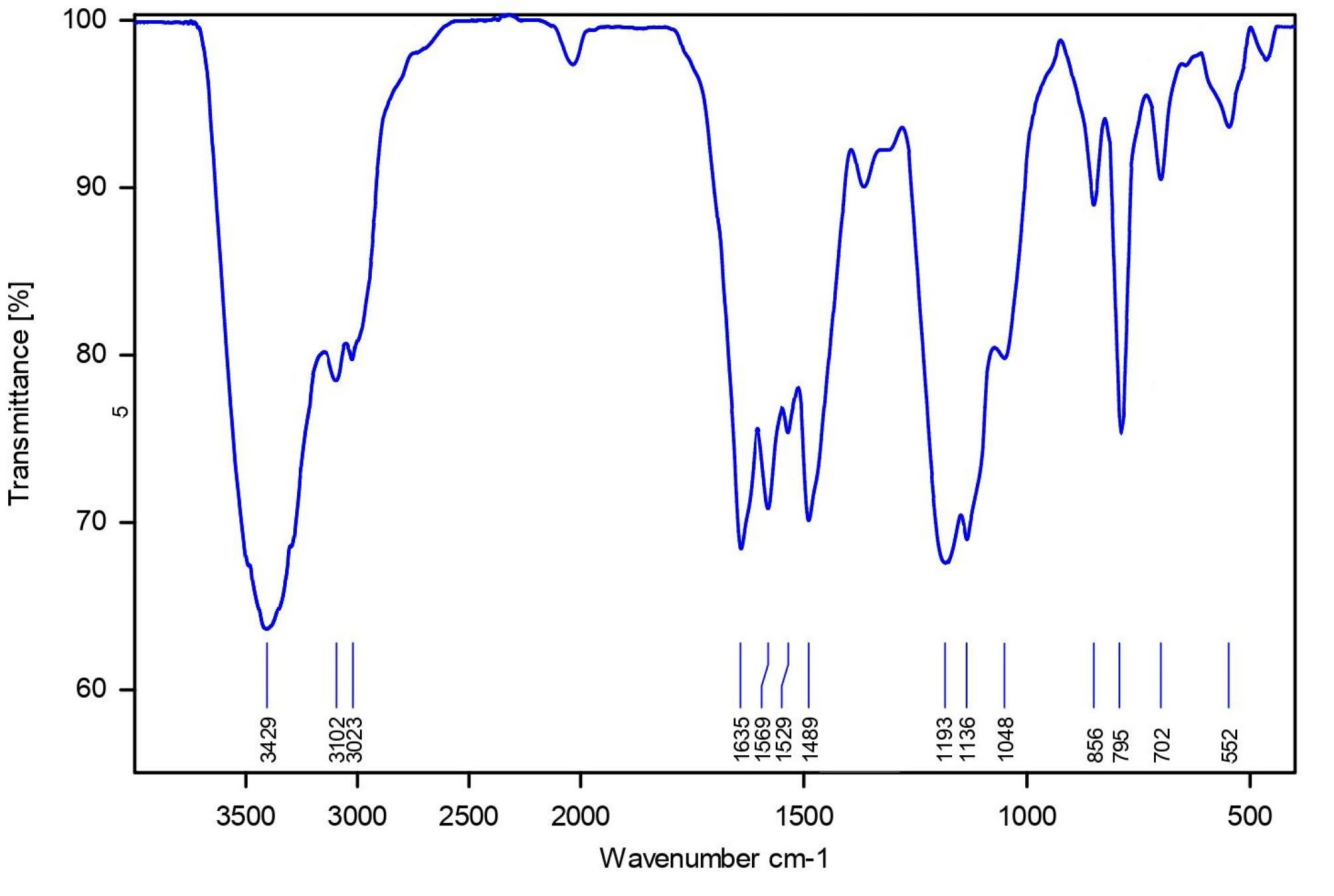
FT-IR spectrum of PDSPTCF.

**Fig. 4:**
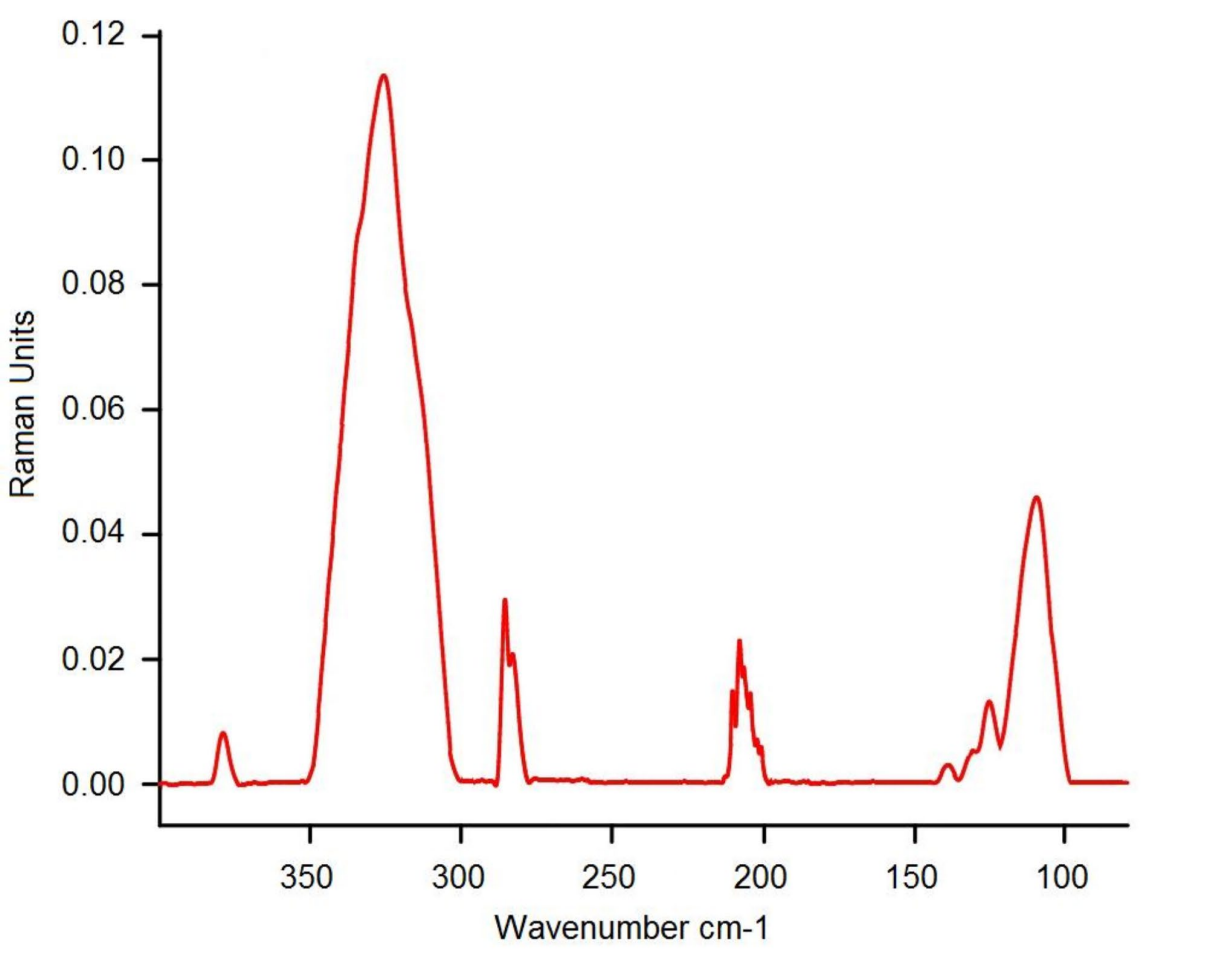
Raman spectrum of PDSPTCF.

In Table 1, the most important absorptions of bonds and functional groups in FT-IR (entries 1 to 9) and Raman (entries 10 and 11) spectra of PDSPTCF are listed (Figs. 3 and 4). These data support the creation of new bonds and the formation of PDSPTCF organic–inorganic salt skeleton^54–57^. It is worth mentioning that the purpose of recording the Raman spectrum of the catalyst was to identify the vibrational frequencies of Fe-Cl and Cl-Fe-Cl in tetrachloroferrate (III) ion, which appear in the range of 100–400 cm^-1^^57^.

**Table 1:** The FT-IR (entries1 to 9) and Raman (entries 10 and 11) data of PDSPTCF.

Entry	Adsorption (cm^−1^)	Bond or functional group
1	552	S–O bending^54^
2	795	C–H out-of-plane^55^ =
3	856	N–S stretching^54^
4	1048	S–OH bending^54^
5	1193 and 1136	S–O asymmetric and symmetric stretching^54^
6	1569, 1489, ~ 1400	C = C stretching^56^
7	1635	C = N stretching^56^
8	3102	C–H symmetric stretching^56^
9	2600–3550	O–H stretching vibration of SO_3_H^54^
10	378 and 330	Fe–Cl stretching of tetrachloroferrate^57^
11	130 and 109	Cl–Fe–Cl twist of tetrachloroferrate^57^

**Fig. 5:**
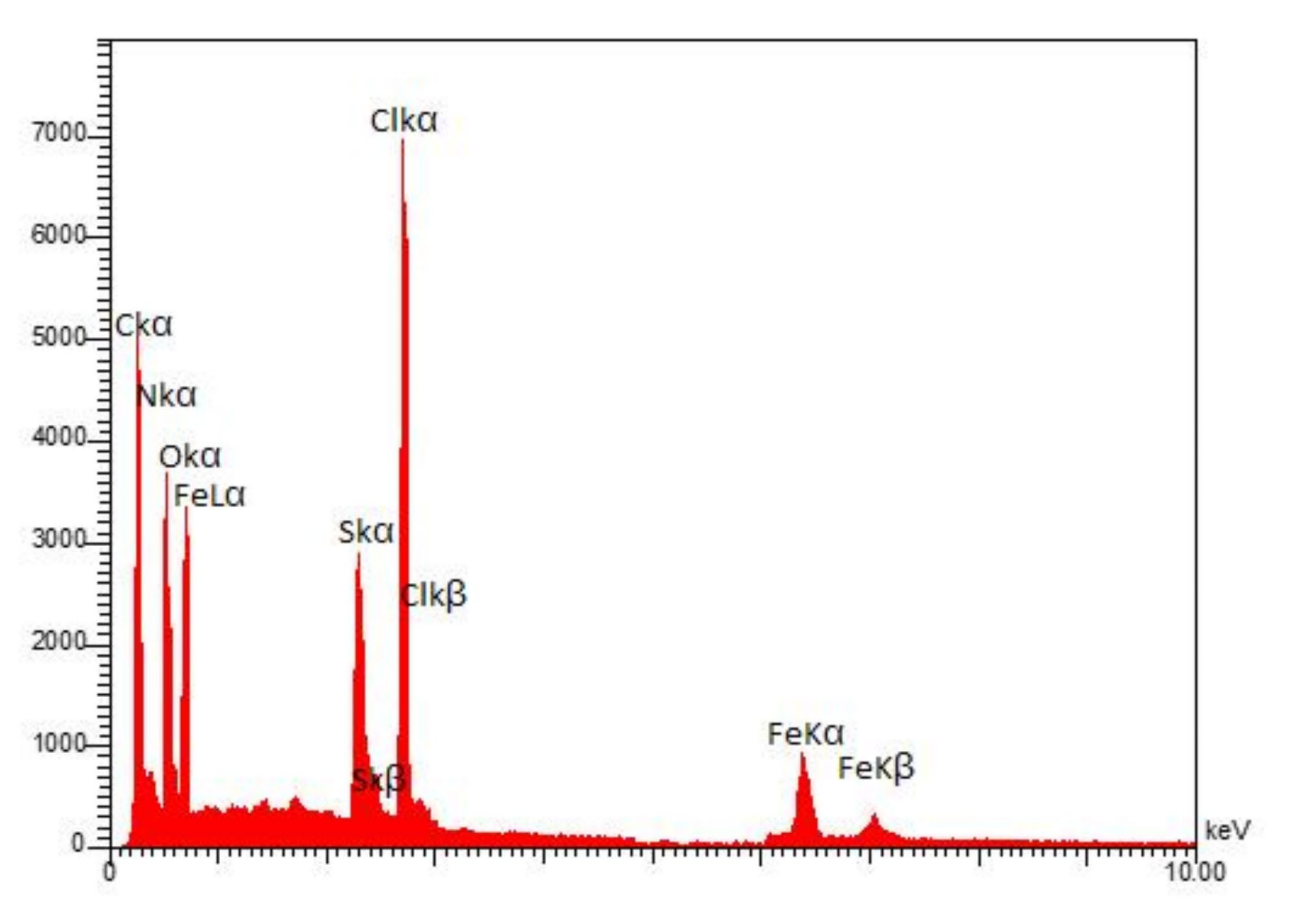
EDS spectrum of PDSPTCF.

**Fig. 6:**
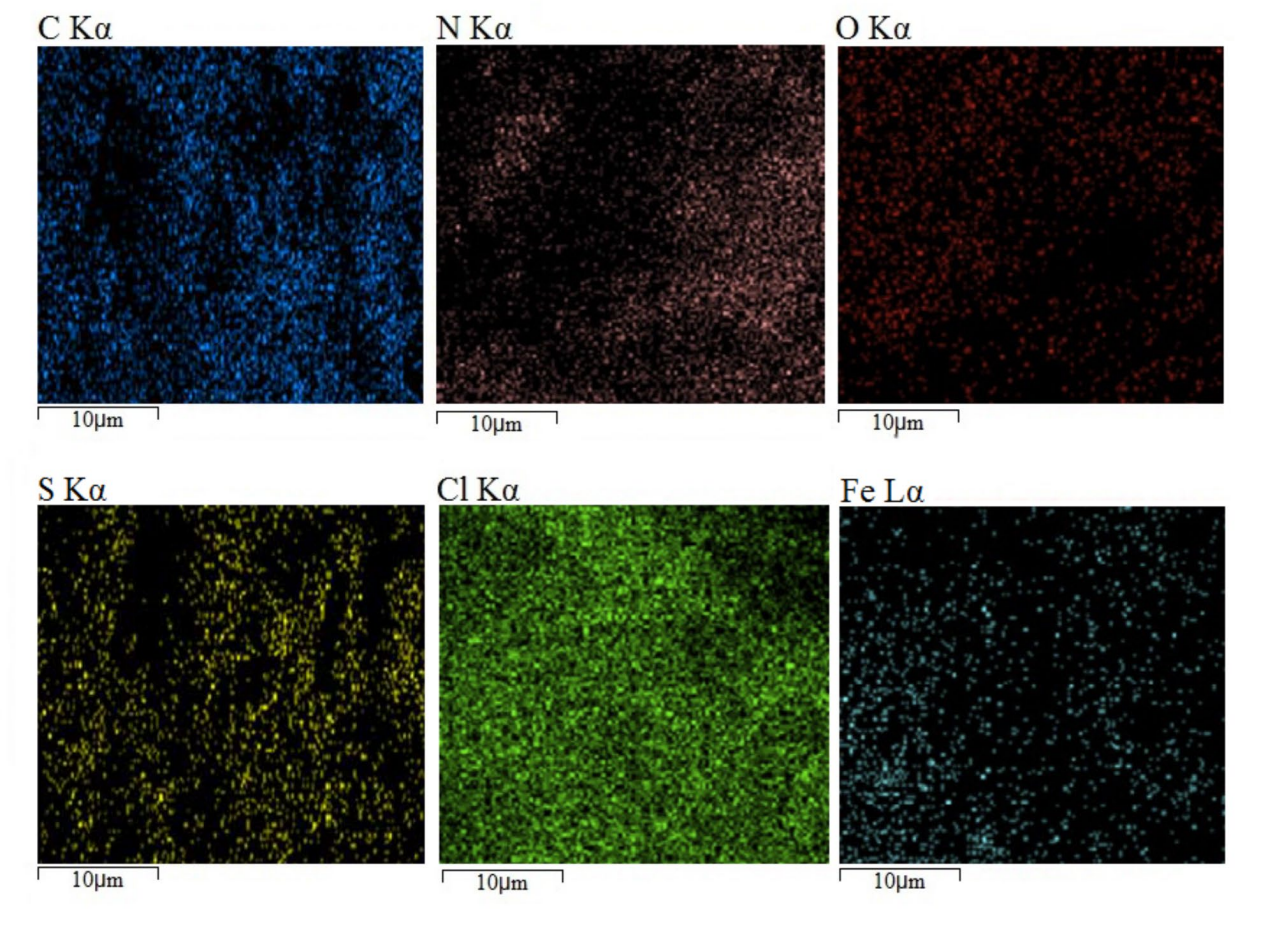
Elemental mapping analysis of PDSPTCF.

The presence of all elements constituting the skeleton of PDSPTCF (O, S, N, C, Cl and Fe) was confirmed by the EDS technique without observing additional elements (Fig. 5). The weight percents of O, S, N, C, Cl and Fe were 12.01, 8.10, 3.44, 24.12, 35.88 and 16.45, respectively (Table 2). Further, elemental mapping analysis (with the support of the EDS technique results) proved the presence of the mentioned elements in the organic–inorganic compound salt structure (Fig. 6).

**Table 2:** Quantitative results of the EDS analysis of PDSPTCF.

Element	O	S	N	C	Cl	Fe
W%	12.01	8.10	3.44	24.12	35.88	16.45

**Fig. 7:**
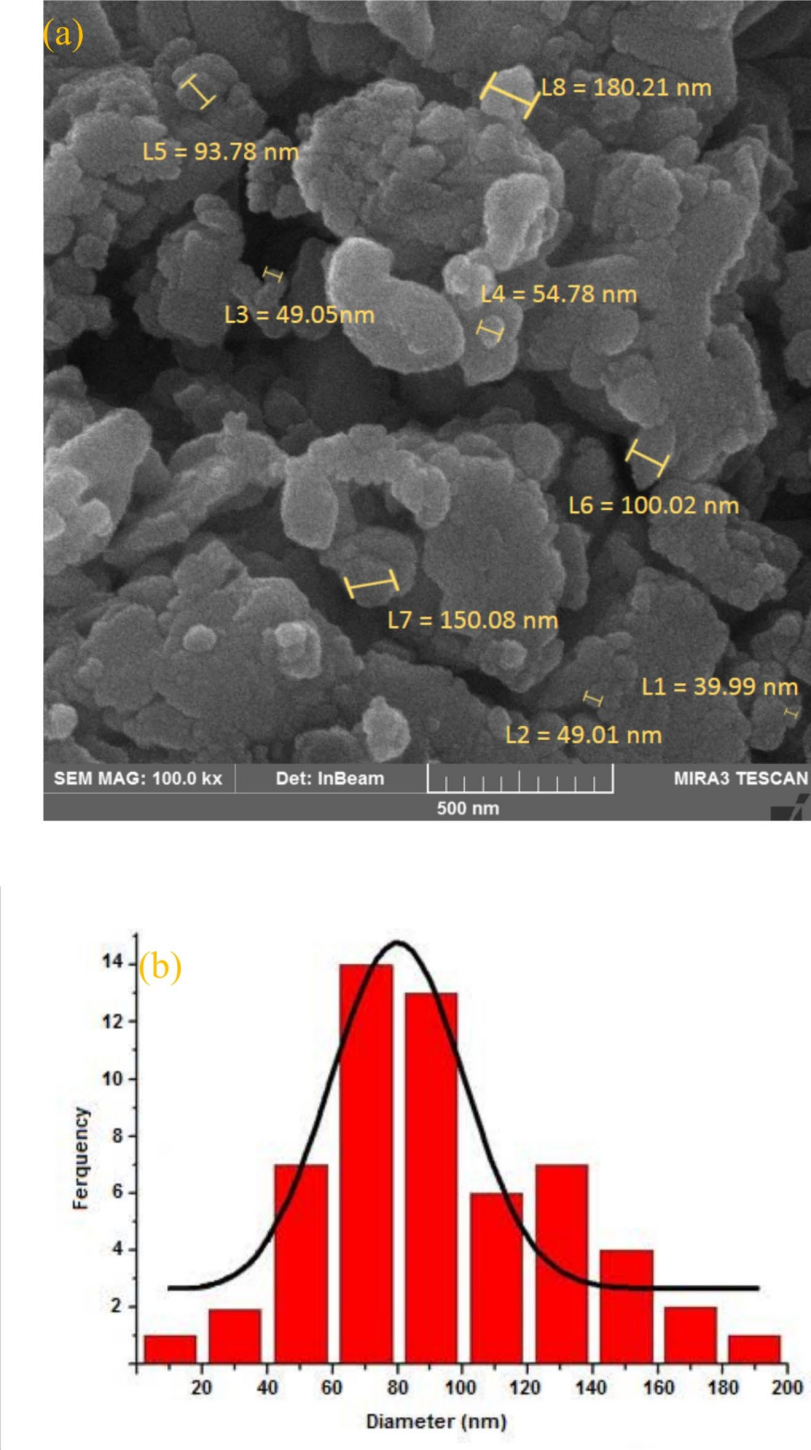
FE-SEM images (**a**) and particle size distribution (**b**) of PDSPTCF.

Based on FE-SEM images of PDSPTCF (Fig. 7a), the size of most of our hybrid salt particles was in the range of 50–180 nm, and only some particles had sizes less than 50 nm. Also, most PDSPTCF particles were in the shape of connected quasi-spherical clusters, and only a few of them were in the form of agglomerates of amorphous pieces. Moreover, the particle size distribution histogram of PDSPTCF revealed that the particles of this hybrid salt have a size in the range of 20 to 200 nm and a mean diameter of 92.45 nm (Fig. 7b).

**Fig. 8:**
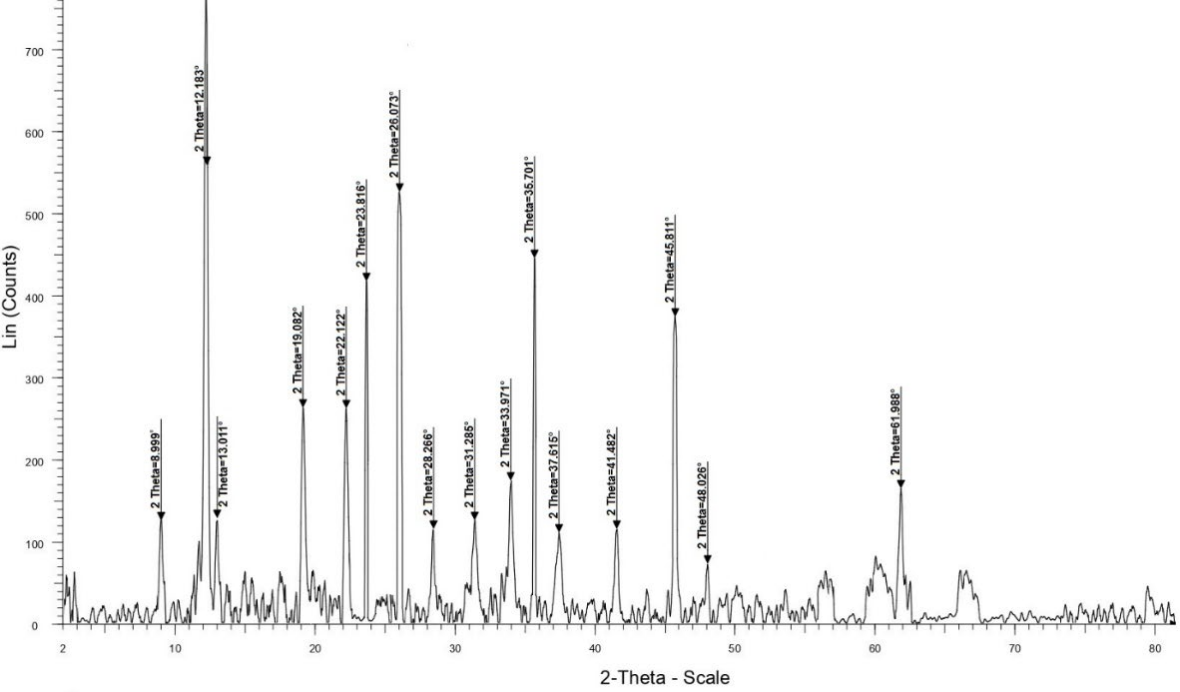
XRD pattern of PDSPTCF.

XRD pattern of the PDSPTCF (Fig. 8) reflected a few relatively broad peaks at 2θ = 67.47°-66.01°, 61.21°-59.23°, 57.09°-56.01°, and several diffraction lines at Bragg angles of 61.98°, 48.02°, 45.81°, 41.48°, 37.61°, 35.70°, 33.97°, 31.28°, 28.26°, 26.07°, 23.81°, 22.12°, 19.08°, 13.01°, 12.18°, 8.99. Based on these data, only some of the catalyst particles have an amorphous form and most of them have a crystalline nature^21,58,59^. The crystalline and amorphous percentages of PDSPTCF hybrid salt were 79.88% and 20.12%, respectively. The literature supports these interpretations^60^.

**Fig. 9:**
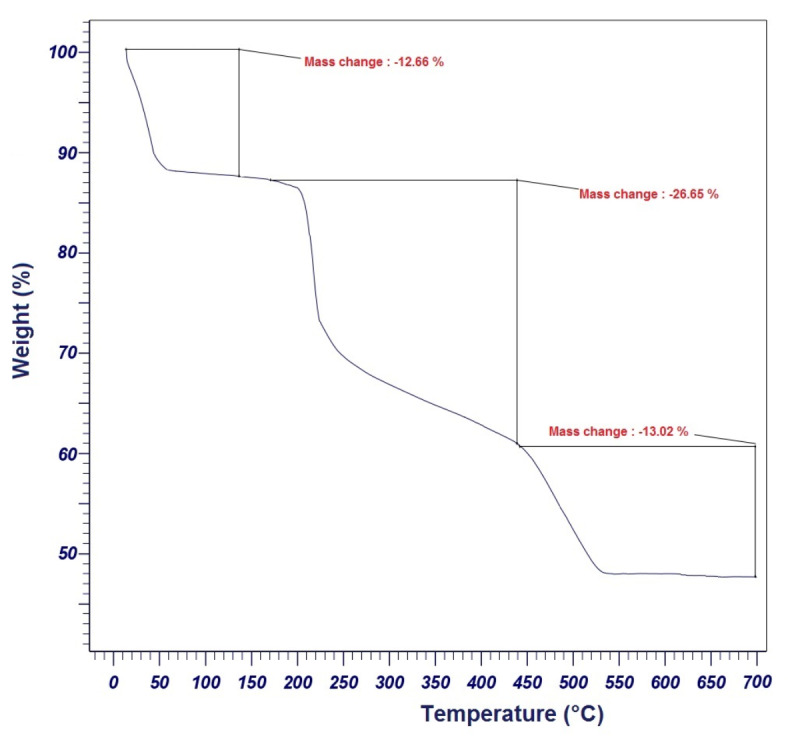
TG diagram of PDSPTCF.

The thermal stability of PDSPTCF was evaluated by TGA in the range of 25–700 °C (Fig. 9). The TGA graph reflected three stages of weight loss. The 12.66% initial weight loss below about 170 °C can be assigned to the evaporation of H_2_O and other solvents adsorbed by the PDSPTCF. Two more consecutive decompositions (in the range of 170 to 440 °C and 440 to 700 °C) led to a weight loss of 39.67% of the hybrid salt weight, which is due to the oxidation of tetrachloroferrate by SO_3_ oxygen, the transformation of tetrachloroferrate into iron chloride, and the decomposition of other organic parts. The literature supports these interpretations^13,62,63^. Reasonable weight loss (52.33% decomposition) of PDSPTCF organic–inorganic hybrid salt showed that the catalyst has good thermal stability. Therefore, it can be used as a catalyst for organic conversions that require harsh temperature conditions.

**Fig. 10:**
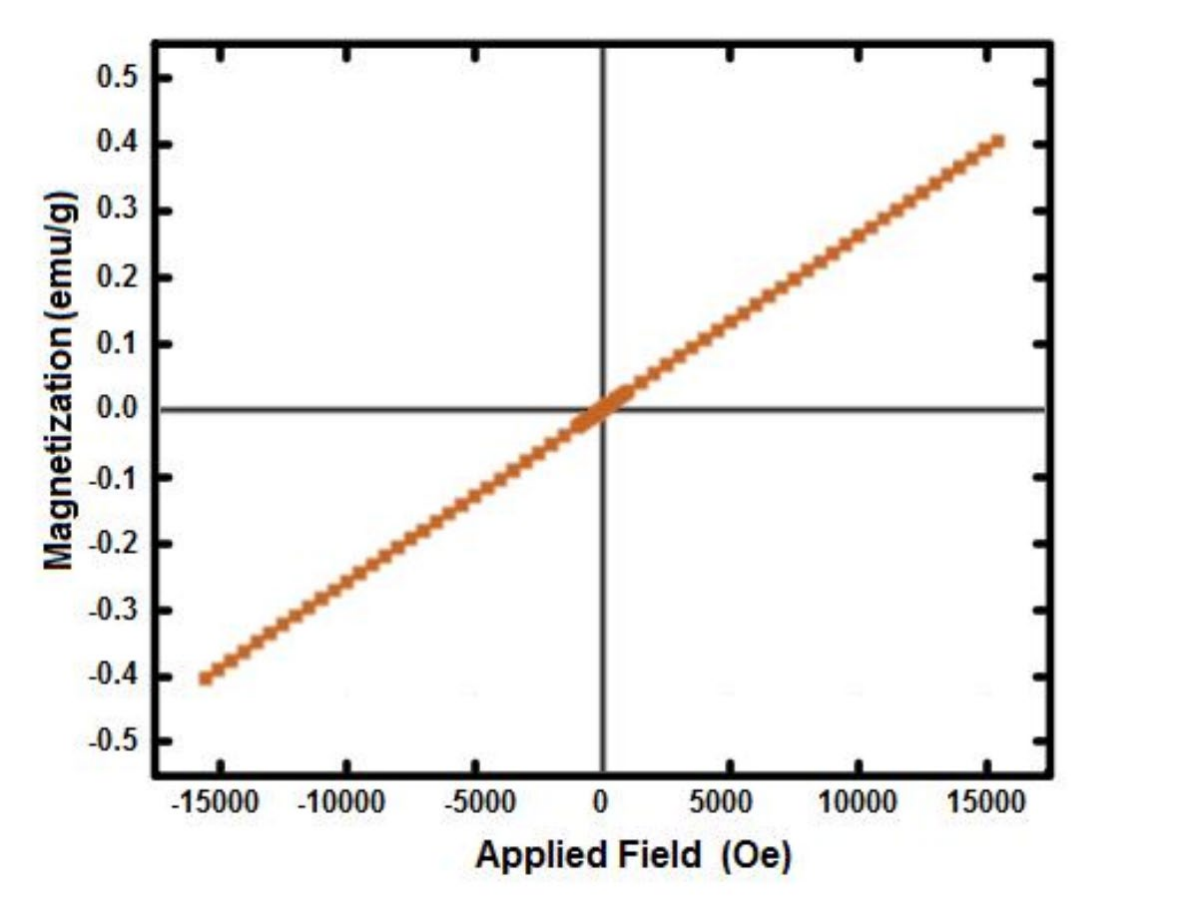
VSM diagram of PDSPTCF.

The VSM diagram of PDSPTCF hybrid salt was recorded by a vibrating sample magnetometer at a temperature of 25 °C (Fig. 10). A linear plot with a magnetic moment of 0.04102 emu g^−1^ magnetic fields was found for this salt, which indicates the paramagnetic property^63,64^. However, this magnetic property was insufficient to separate the catalyst from the reaction mixture with a conventional magnet^56^. This issue is probably due to the tetrachloroferrate anion core being covered with the organic and inorganic moieties of the hybrid salt cation^64–66^.

### PDSPTCF catalytic activity

**Fig. 11:**
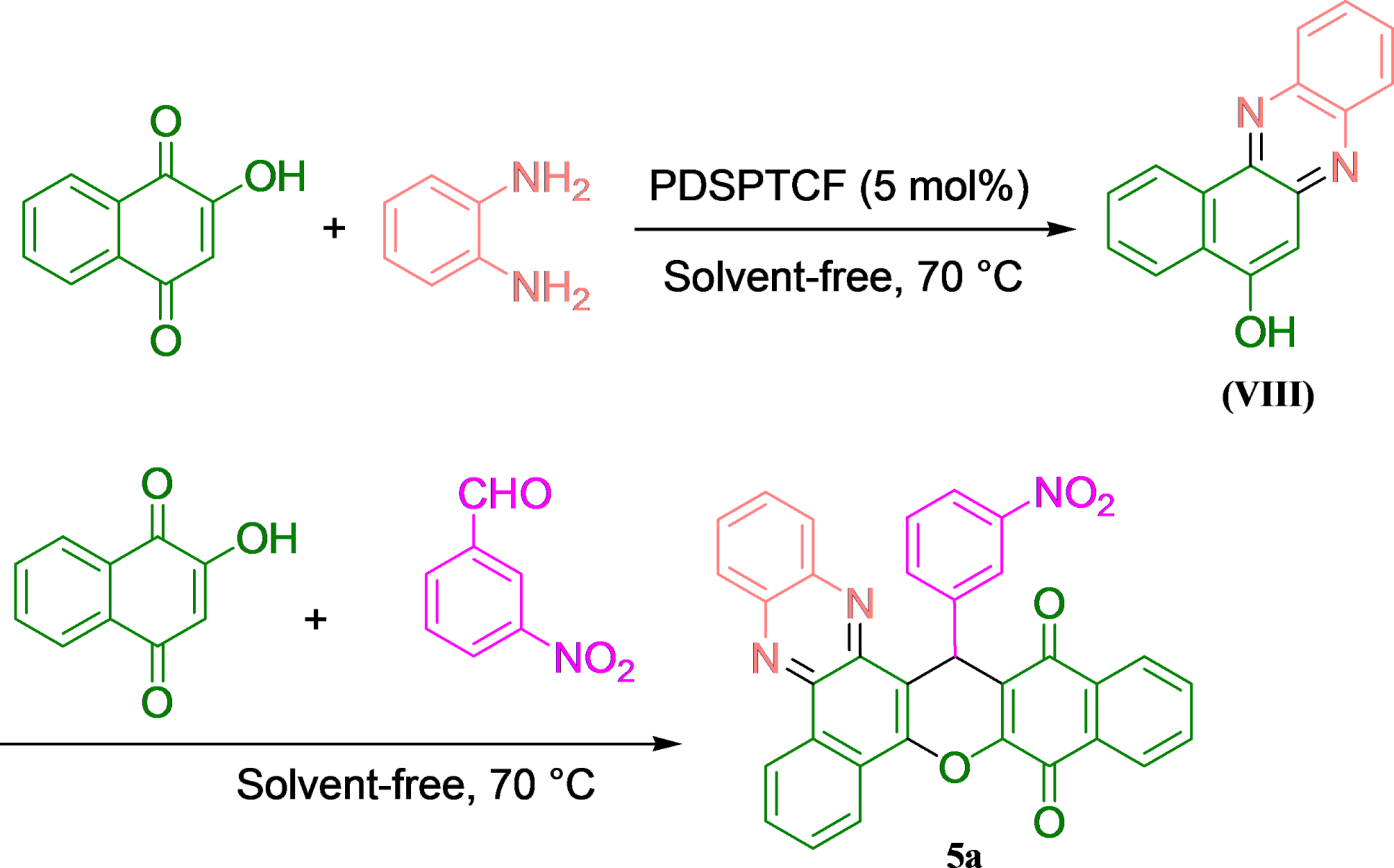
The model reaction.

After the successful synthesis and identification of PDSPTCF, the next step was to evaluate its performance in producing benzo[a]benzo[6,7]chromeno[2,3-*c*]phenazines. To do this, one-pot MDR reaction between 2-hydroxynaphthalene-1,4-dione (2 eq.), benzene-1,2-diamine (1 eq.), and 3-nitrobenzaldehyde (1 eq.) were chosen as model (Fig. 11). First, the progress of this reaction was checked at a temperature of 130°C under the condition of free catalysis, which was accompanied by complete failure (Table 3, entry 1). Next, the reaction was evaluated with 7 mol% of catalyst in different solvent environment {including ethanol (EtOH), ethyl acetate (EtOAc), acetonitrile (CH_3_CN), dimethyl sulfoxide (DMSO) and chloroform (CHCl_3_)} (Table 3, entries 2–6), which did not lead to the desired yield after 120 min. With parent PDSPTCF as a catalyst, the reaction did not progress significantly (Table 3, entries 7–9). Also, monitoring the progress of the reaction with PDSPDC ionic liquid as a catalyst resulted in a not-so-impressive yield of 82% in 12 min (Table 3, entry 10). Finally, by screening various factors in the progress of the model reaction in the solvent-free environment (Table 3, entries 11–14), it was found that using a temperature of 70 °C with 5 mol% PDSPTCF is the optimal condition for the aforementioned condensation (Table 3, entry 11, Time: 12 min, Yield: 98%).

**Table 3:** Optimization of various parameters on the reaction of 2-hydroxynaphthalene-1,4-dione with benzene-1,2-diamine, and 3-nitrobenzaldehyde (Fig. 11).

Entry	Catalyst	Solvent (2 mL)	Temp. (°C)	Catalyst amount (mol%)	Time (min)	Yield^a^ (%)
1	–	–	130	–	120	–^b^
2	PDSPTCF	EtOH	Reflux	7	120	85
3	PDSPTCF	EtOAc	Reflux	7	120	84
4	PDSPTCF	CH_3_CN	Reflux	7	120	88
5	PDSPTCF	DMSO	120	7	120	90
6	PDSPTCF	CHCl_3_	Reflux	7	120	75
7	FeCl_3_	–	90	5	30	32
8	Chlorosulfonic acid	–	90	5	30	45
9	1,4-bis(pyrid-4-yl)benzene	–	90	5	30	trace
10	PDSPDC	–	70	5	12	82
11	PDSPTCF	–	70	5	12	98
12	PDSPTCF	–	70	3	20	70
13	PDSPTCF	–	70	7	12	98
14	PDSPTCF	–	90	5	12	98
15	PDSPTCF	–	50	5	12	88

^a^Isolated yield.

^b^No reaction.

To assess the scope and influence of PDSPTCF in preparing benzo[a]benzo[6,7]chromeno[2,3-*c*]phenazines, the MDR reaction between 2-hydroxynaphthalene-1,4-dione, benzene-1,2-diamine with various aromatic aldehydes (with activating and deactivating substitutions of the aromatic ring) was probed under the best circumstances. The acquired reaction yields, times, turnover number frequencies (TONs), and turnover frequencies (TOFs) are provided in Table 4. In all instances, the corresponding benzo[*a*]benzo[6,7]chromeno[2,3-*c*]phenazines were achieved in short times (12 to 20 min), high yields (88% to 98%), and good TON (17.60 to 19.60) and TOF (0.88 to 1.63 min^−1^). Moreover, purification of all derivatives was performed without using column chromatography and only by recrystallization from 95% EtOH. In the presence of aliphatic aldehydes e.g. *n*-octanal and *n*-heptanal, the expected product under optimal conditions was not received even after 60 min (Table 4, entries 13a, 14a).

**Table 4:** Producing benzo[*a*]benzo[6,7]chromeno[2,3-*c*]phenazines catalyzed by PDSPTCF.


Product	R	Time (min)	Yield (%)^a^	TON	TOF (min^−1^)	M.p. (°C) Found (Reported)
1a	2-Cl-C_6_H_4_	14	95	19.00	1.35	335–337 (336–338)^53^
2a	4-Cl-C_6_H_4_	12	97	19.40	1.61	325–327 (324–326)^53^
3a	2,4-Cl_2_-C_6_H_3_	17	93	18.60	1.09	330–332 (330–332)^53^
4a	2-O_2_N-C_6_H_4_	13	98	19.60	1.50	294–296 (293–295)^52^
5a	3-O_2_N-C_6_H_4_	12	98	19.60	1.63	368–370 (368–370)^53^
6a	4-O_2_N-C_6_H_4_	12	98	19.60	1.63	272–274 (274–275)^50^
7a	3-CN-C_6_H_4_	13	97	19.40	1.49	286–288 (286–288)^51^
8a	4-OH-C_6_H_4_	15	92	18.40	1.22	300–302 (301–303)^51^
9a	2-OH-5-Br-C_6_H_4_	20	88	17.60	0.88	358–360 (359–361)^53^
10a	4-MeO-C_6_H_4_	16	94	18.80	1.17	340–342 (341–342)^53^
11a	4-Me-C_6_H_4_	14	96	19.20	1.37	331–333 (332–334)^50^
12a	3,4-(MeO)_2_-C_6_H_3_	16	92	18.40	1.15	318–320 (320–321)^51^
13a	CH₃(CH₂)₆^b^	60	–^c^	–	–	–
14a	CH₃(CH₂)_5_^d^	60	–^c^	–	–	–

^a^Isolated yield.

^b^Full name of aldehyde: *n*-Octanal.

^c^No reaction.

^d^Full name of aldehyde: *n*-Heptanal.

**Fig. 12:**
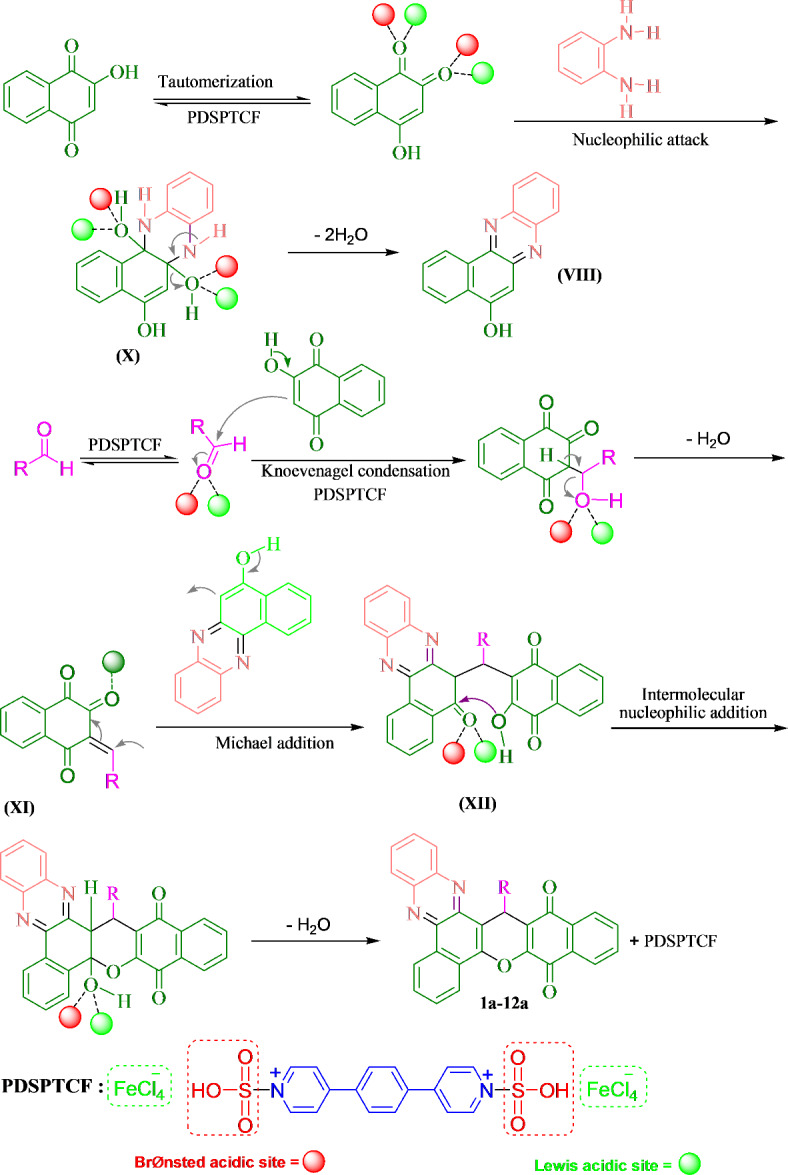
Reaction mechanism in the synthesis of benzo[*a*]benzo[6,7]chromeno[2,3-*c*]phenazines.

Based on the literature^50,53,65^ and Lewis–Brønsted acidity of PDSPTCF, a logical route for the preparation of benzo[*a*]benzo[6,7]chromeno[2,3-*c*]phenazines was proposed (Fig. 12). In the first step, 2-hydroxynaphthalene-1,4-dione performs the tautomerization process in the vicinity of the catalyst and turns into 4-hydroxynaphthalene-1,2-dione tautomer, then the carbonyl groups of this tautomer are activated by the Lewis and Brønsted acidic moieties of the catalyst (tetrachloroferrate and SO_3_H). Next, NH_2_ groups of benzene-1,2-diamine performed a nucleophilic attack to the activated carbonyl groups to give **X**. The Lewis and Brønsted acidic positions of the hybrid material expedite eliminating a water molecule from **X** to acquire **VIII**. On the other hand, the acidic moieties of PDSPTCF activates aldehyde; then, nucleophilic attack of 2-hydroxynaphthalene-1,4-dione (second mole) to the activated aldehyde affords **XI** (Knoevenagel condensation). Michael-type addition of **VIII** to the activated **XI** (via the tetrachloroferrate and SO_3_H groups of the catalyst) provides **XII**. Intermolecular nucleophilic addition of the hydroxyl group of **XII** to its activated carbonyl group (via acidic sites of PDSPTCF), and removal of a water molecule gives benzo[*a*]benzo[6,7]chromeno[2,3-*c*]phenazines (**1a-12a**). Notably, Lewis acidity of FeCl_4_ˉ containing catalysts has been referred in the previous sources^67^. The high effectiveness of PDSPTCF can be associated to its Lewis–Brønsted acidic nature and owning two positions of each acid.

### PDSPTCF reusability

**Fig. 13:**
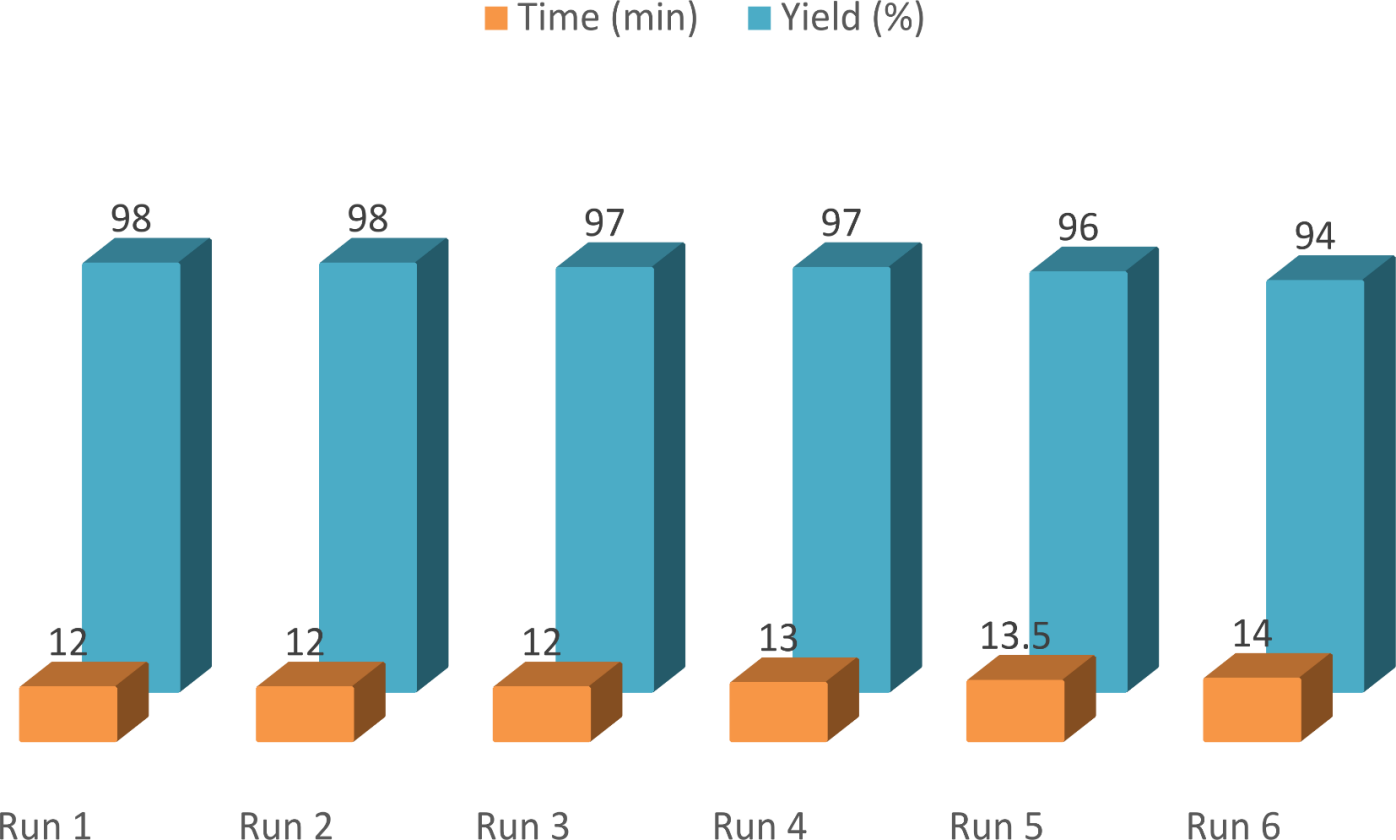
Results of six PDSPTCF recycling cycles in the synthesis of product **5a.**

The reusability of PDSPTCF was investigated through the condensation leading to the production of product **5a** under optimal reaction conditions. To do this, after the completion of the reaction, the catalyst was separated according to the technique described in the experimental section and prepared for the next run. Figure 13 shows the catalytic performance of PDSPTCF after six cycles as a function of time and efficiency. The findings revealed that the catalytic activity of our organic–inorganic hybrid salt decreased slightly during six consecutive cycles (4%).

**Fig. 14:**
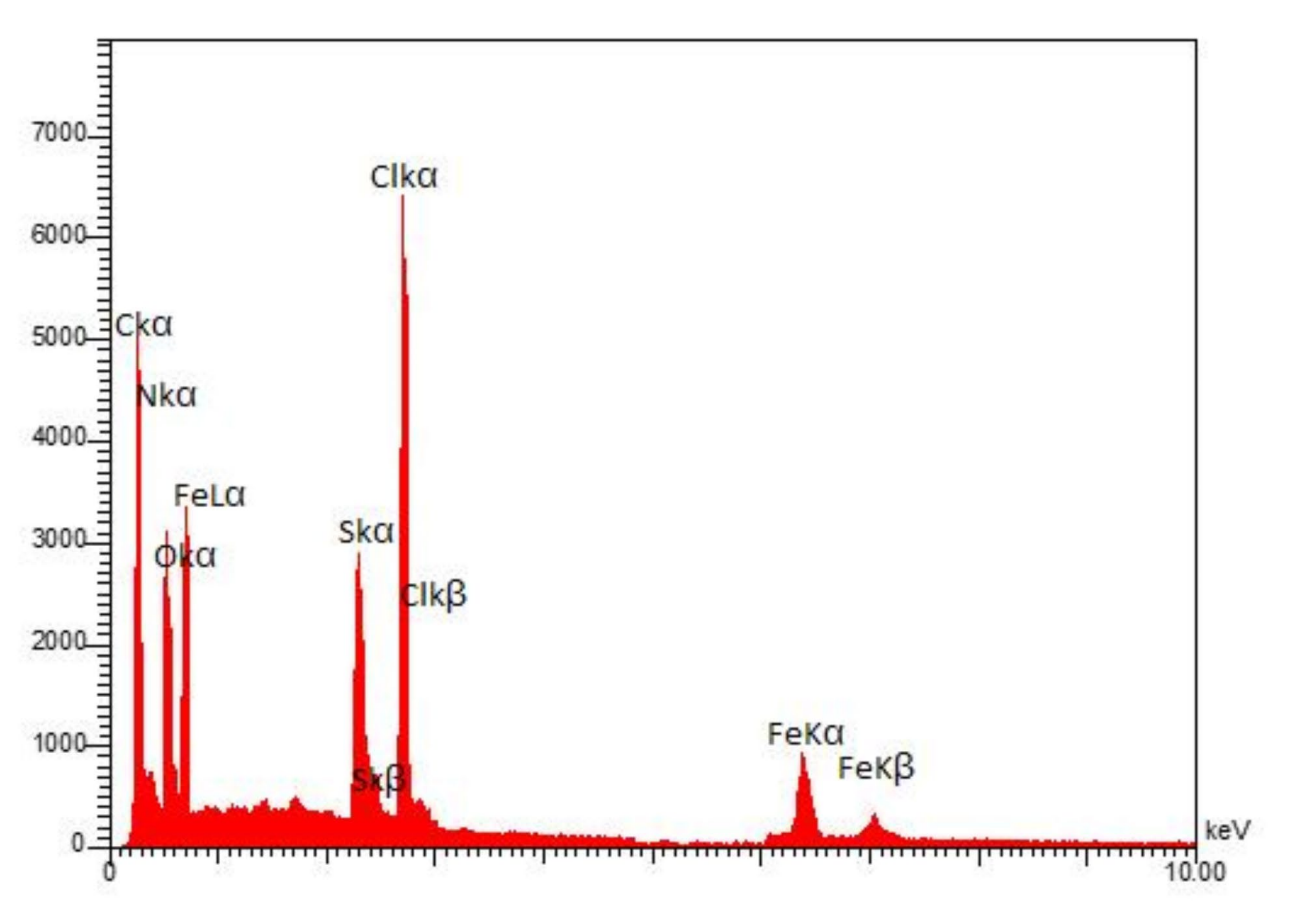
EDS image of retrieved PDSPTCF.

**Fig. 15:**
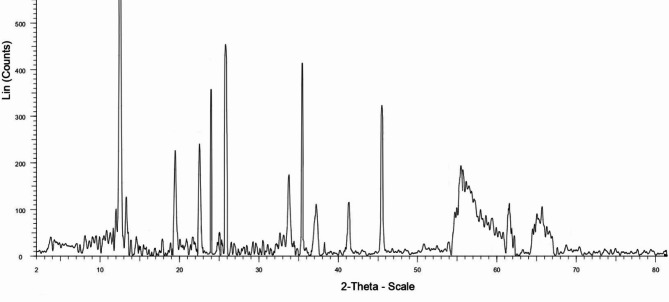
XRD pattern of retrieved PDSPTCF.

In the following, the non-leaching of PDSPTCF (especially the metal part: iron) was investigated through ICP-OES and hot filtration methods, and its structural changes using EDS (Fig. 14) and XRD (Fig. 15) techniques after the sixth recycling of derivative **5a**. Based on the ICP-OES method, the amount of iron in the reused and fresh catalyst was 1.68 × 10^–3^ mol g^−1^ and 1.71 × 10^–3^ mol g^−1^, respectively. These data confirm partial leaching of the PBPBPMF catalyst. The results of this study were confirmed through the hot filtration test because the percentage of product **5a** was 60% in half the reaction time (6 min). After removing the catalyst, the reaction mixture without it produced only 65% of the product **5a** in another six minutes, which means that a heterogeneous mechanism is at work throughout the recycling process.

The evaluation of EDS (Fig. 14) and XRD (Fig. 15) images of the spent catalyst with its fresh sample indicated that these images matched the corresponding analysis of the fresh sample of PDSPTCF. However, in the XRD pattern of the used catalyst, it was evident that the crystalline character of the catalyst was reduced (due to the removal of peaks 48.02°, 31.28°, 28.26° and 8.99° as well as increasing the intensity of the broad peaks related to the amorphous nature of the catalyst in the region 2θ = 54° to 67° in Fig. 8 compared to Fig. 15). The sum of the above observations led us to conclude that the relative decrease in the activity of the PDSPTCF hybrid salt is owing to the loss of about 2–5% during the 6 cycles of the recovery process and possibly the reduction of its crystalline nature.

### Comparison

The catalytic activity of PDSPTCF versus other catalysts in the synthesizing benzo[a]benzo[6,7]chromeno[2,3-*c*]phenazines was analyzed by evaluating factors such as reaction time, the amount of catalyst consumed, product yield, TOF, and the benignity of the reaction environment (i.e., reaction medium with solvent against without solvent). To achieve this goal, the condensation leading to the synthesis of 17-(3-nitrophenyl)-11*H*-benzo[*a*]benzo[6,7]chromeno[2,3-*c*]phenazine-11,16(17*H*)-dione (**5a**) was deemed as a benchmark, and the outcomes are presented in Table 5. Examination of the table data reveals the superior catalytic performance of PDSPTCF compared to competitors.

**Table 5:** Catalytic activity of PDSPTCF vs. other available catalysts in the synthesizing benzo[*a*]benzo[6,7]chromeno[2,3-*c*]phenazines.

Catalyst	Conditions	Time (min)	Yield^a^ (%)	TOF (min^−1^)	Ref
L-proline (20 mol%)	H_2_O, Microwave, 180 W, max. 70 °C	10	95	0.47	^49^
Zr-guanine-MCM-41 (0.30 mol%)	PEG-400, 100 °C	120	90	2.50	^50^
γ‐Fe_2_O_3_@SiO_2_‐SCH_2_CO_2_H (0.03 g)	H_2_O/EtOH (1:1), 70 °C	120	94	–^b^	^51^
Ni-Gly-isatin@boehmite (1.15 mol%)	PEG-400, 80 °C	300	90	0.26	^52^
*p*-Toluenesulfonic acid (20 mol%)	PEG-400, 80 °C	120	91	0.03	^53^
PDSPTCF (5 mol%)	Solvent-free, 70°C	12	98	1.63	–

^a^Isolated yield.

^b^ In this work, mol% of the catalyst has not been reported; thus, we could not calculate TOF of the catalyst.

In another study, the catalytic performance of PDSPTCF was studied in comparison with other Brønsted-Lewis acid catalysts available in the literature for the synthesis of heterocyclic compounds by evaluating the factors shown in Table 6. The results showed that PDSPTCF is superior to other catalysts in most of the compared cases.

**Table 6:** Comparison of catalytic performance of PDSPTCF against other Brønsted–Lewis acidic catalysts available in the literature for the synthesis of heterocyclic compounds.

Catalyst	Product	Conditions	Time (min)	Yield^a^ (%)	TOF (min^−1^)	Ref
Ni-DDIA (1.5 mol%)		Solvent-free, 80 °C	30	88.3–93.2	1.96–2.07	^68^
[Piper-(SO_3_H)_2_].[FeCl_4_]_2_ (10 mol%)		CH_2_Cl_2_, reflux	120–180	74–89	0.14–0.04	^69^
Hnmp/ZnCl_3_ (7.35 mol%)		Solvent-free, 100 °C	30–50	78–97	0.21–0.39	^70^
bmim[FeCl_4_] (5 mol%)		Solvent-free, 40 °C	90–210	86–95	0.08–0.20	^71^
[AcMIM]FeCl_4_ (20 mol%)		Solvent-free, 60 °C	60	91–94	0.075–0.078	^72^
MIL-101(Cr)-NH-CO-Pr-COOH (4.5 mol%)		Solvent-free, 80 °C	30–240	70–93	0.07–0.83	^73^
PDSPTCF (5 mol%)		Solvent-free, 70 °C	12–20	88–98	0.88–1.63	–

^a^Isolated yield.

## Conclusions

In summary, a new organic–inorganic salt as a new Brønsted-Lewis acid catalyst namely 4,4'-(1,4-phenylene)di(sulfonic)pyridinium tetrachloroferrate (PDSPTCF) was synthesized and characterized by a wide range of physicochemical methods. This hybrid salt having two types of acid sites (two tetrachloroferrate anions as Lewis acidic sites and two SO_3_H groups as Brønsted acidic sites) was successfully used to catalyze the reaction leading to the production of benzo[*a*]benzo[6,7]chromeno[2,3-*c*]phenazines (**1a**-**12a**, 70 °C, solvent-free). PDSPTCF was consumed up to six sequential cycles with a medium yield of 96.66%, and the durability of its skeleton after recovery was reviewed by EDX and XRD techniques. This protocol has the following privileges: short reaction times (12 to 20 min), effectuality, high yields (88% to 98%), generality, usage of solvent-free environment, good TON and TOF, simplicity (workup and purification of the products), using of a Brønsted–Lewis acid catalyst, compliance with green chemistry standards, supremacy of PDSPTCF over many published catalysts, and easy separation of the catalyst from the reaction mixture. Therefore, our vision for future work will be to further optimize the catalytic system in other types of organic reactions and investigate its applicability on an industrial scale.

## Data Availability

The data that support the findings of this study are available on request from the corresponding author.
